# Supplementary material for: Antiviral efficacy of favipiravir against Zika and SARS-CoV-2 viruses in non-human primates
Source: Nat Commun. 2022 Aug 30;13:5108. doi: 10.1038/s41467-022-32565-w (PMC9427089; doi:10.1038/s41467-022-32565-w)
Supplement: Supplementary file 1 — Supplementary information [file 41467_2022_32565_MOESM1_ESM.pdf]

Antiviral efficacy of favipiravir against Zika and SARS-CoV-2 viruses in non-human primates

Supplementary information

Supplementary Tables

Supplementary Table I. Animal description

GROUP Name	Code	Sex	Date of Birth
**ZIKV CTRL**	MF1	male	16/12/2013
**ZIKV CTRL**	MF3	male	31/12/2012
**ZIKV CTRL**	MF5	male	17/05/2013
**ZIKV CTRL**	MF6	male	28/07/2013
**ZIKV CTRL**	MF7	male	29/11/2013
**ZIKV CTRL**	MF8	male	03/02/2014
**ZIKV 150mg/kg BID**	MF2	male	12/01/2013
**ZIKV 150mg/kg BID**	MF9	male	14/01/2014
**ZIKV 150mg/kg BID**	MF4	male	27/01/2014
**ZIKV 150mg/kg BID**	MF10	male	19/10/2013
**ZIKV 150mg/kg BID**	MF11	male	15/01/2014
**ZIKV 150mg/kg BID**	MF12	male	25/03/2014
**SARS-CoV-2 CTRL**	MF13	female	10/05/2013
**SARS-CoV-2 CTRL**	MF14	female	16/10/2013
**SARS-CoV-2 CTRL**	MF15	female	30/10/2013
**SARS-CoV-2 CTRL**	MF16	female	11/11/2013
**SARS-CoV-2 CTRL**	MF17	female	03/02/2014
**SARS-CoV-2 CTRL**	MF28	male	02/04/2017
**SARS-CoV-2 CTRL**	MF29	female	16/04/2016
**SARS-CoV-2 CTRL**	MF30	male	15/01/2015
**SARS-CoV-2 CTRL**	MF31	female	21/03/2017
**SARS-CoV-2 CTRL**	MF32	male	14/03/2017
**SARS-CoV-2 180mg/kg BID**	MF18	female	12/09/2013
**SARS-CoV-2 180mg/kg BID**	MF19	female	01/11/2013
**SARS-CoV-2 180mg/kg BID**	MF20	female	01/12/2013
**SARS-CoV-2 180mg/kg BID**	MF21	female	14/01/2014
**SARS-CoV-2 180mg/kg BID**	MF22	female	22/02/2014
**SARS-CoV-2 150mg/kg BID**	MF23	female	01/07/2013
**SARS-CoV-2 150mg/kg BID**	MF24	female	12/08/2013
**SARS-CoV-2 150mg/kg BID**	MF25	female	11/09/2013
**SARS-CoV-2 150mg/kg BID**	MF26	female	01/10/2013
**SARS-CoV-2 150mg/kg BID**	MF27	female	19/11/2013
**SARS-CoV-2 150mg/kg BID**	MF38	male	07/12/2016
**SARS-CoV-2 150mg/kg BID**	MF39	male	13/12/2016
**SARS-CoV-2 150mg/kg BID**	MF40	male	11/05/2016
**SARS-CoV-2 150mg/kg BID**	MF41	female	17/04/2016
**SARS-CoV-2 150mg/kg BID**	MF42	male	11/03/2017
**SARS-CoV-2 100mg/kg BID**	MF33	female	08/04/2016
**SARS-CoV-2 100mg/kg BID**	MF34	male	09/05/2016
**SARS-CoV-2 100mg/kg BID**	MF35	female	16/04/2016
**SARS-CoV-2 100mg/kg BID**	MF36	male	06/03/2017

**SARS-CoV-2 100mg/kg BID**	MF37	male	09/03/2017

Supplementary Table II. Names and sequences of primers.

Name	Sequences (5'-3')
RdRp gene / nCoV_IP4	
nCoV_IP4-14059Fw	GGTAACTGGTATGATTTCG
nCoV_IP4-14146Rv	CTGGTCAAGGTTAATATAGG
nCoV_IP4-14084Probe(+)	TCATACAAACCACGCCAGG [5']Fam [3']BHQ-1
E gene / ZIKV	
ZIKE - F	CCGCTGCCCAACACAAG
ZIKE - R	CCACTAACGTTCTTTTGCAGACAT
ZIKE – Probe	AGCCTACCTTGACAAGCAATCAGACACTCAA[5']Fam [3']BHQ-1

Supplementary figures

Figure S1

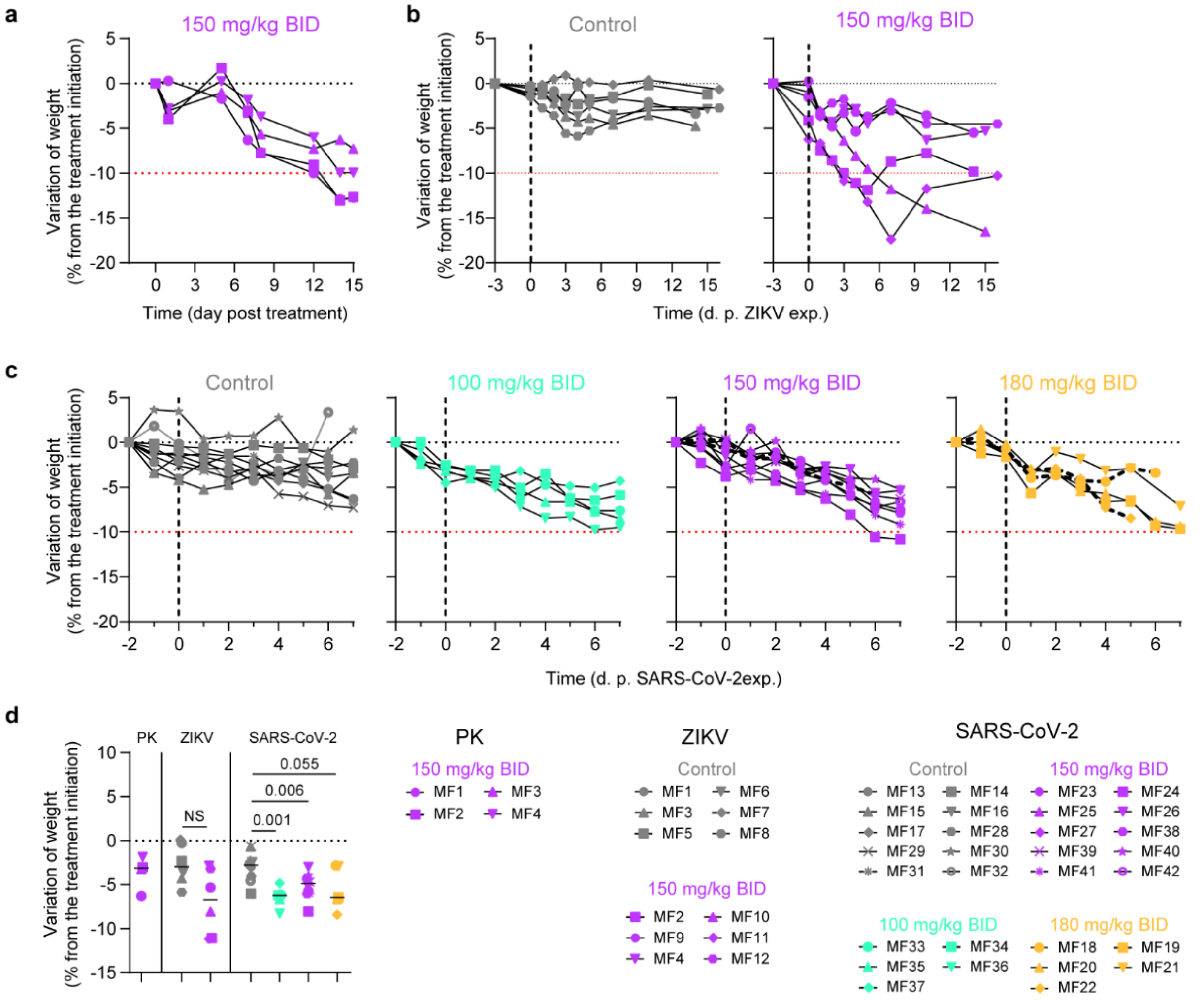

Figure S1. Effect of FPV treatment on body weight. Longitudinal evolution in body weight (relative to value at treatment initiation). a) uninfected animals; b) ZIKV infected animals; c) SARS-CoV-2 infected animals. d) all animals at 7 days post treatment initiation. Median value is indicated by horizontal bar. Grey: untreated; cyan: 100 mg/kg BID; purple: 150 mg/kg BID; yellow: 180 mg/kg BID. Groups were compared between groups using the two-tailed non-parametric Mann-Whitney test with no adjustment for multiple comparison. Source data are provided as a Source Data file.

Figure S2

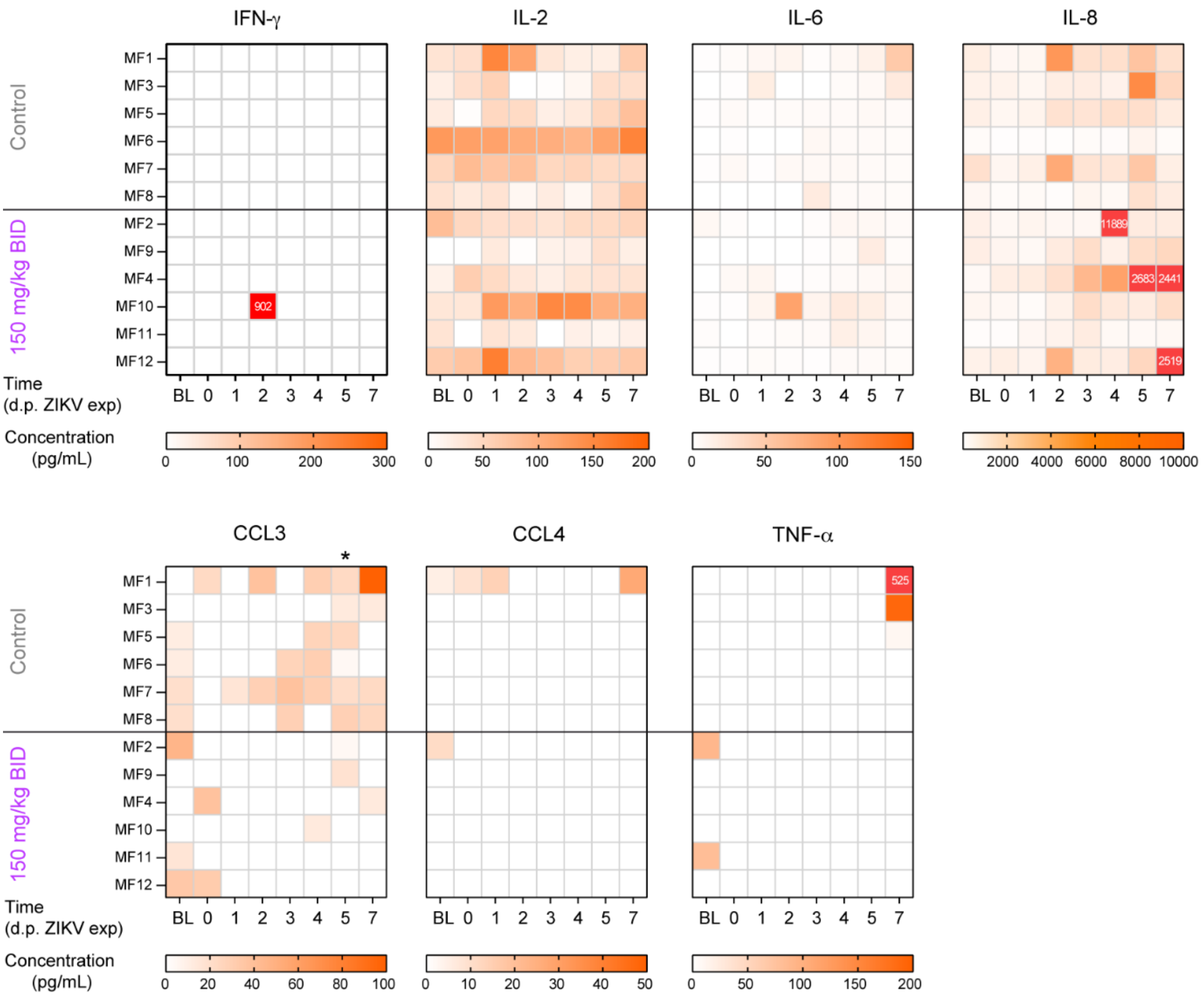

Figure S2. Cytokines and chemokines in the plasma of ZIKV-exposed cynomolgus macaques treated with FPV.

Heatmaps of the plasma concentrations of 7 cytokines and chemokines. Each column represents one cytokine or chemokine; the colour scale (in pg ml−1) is shown at the bottom. The asterisk indicates a significant difference in the concentration of CCL3 at 5 d.p.e. between the control group and the FPV group. Parameters were compared between groups using the two-tailed non-parametric Mann-Whitney test. Source data are provided as a Source Data file.

Figure S3

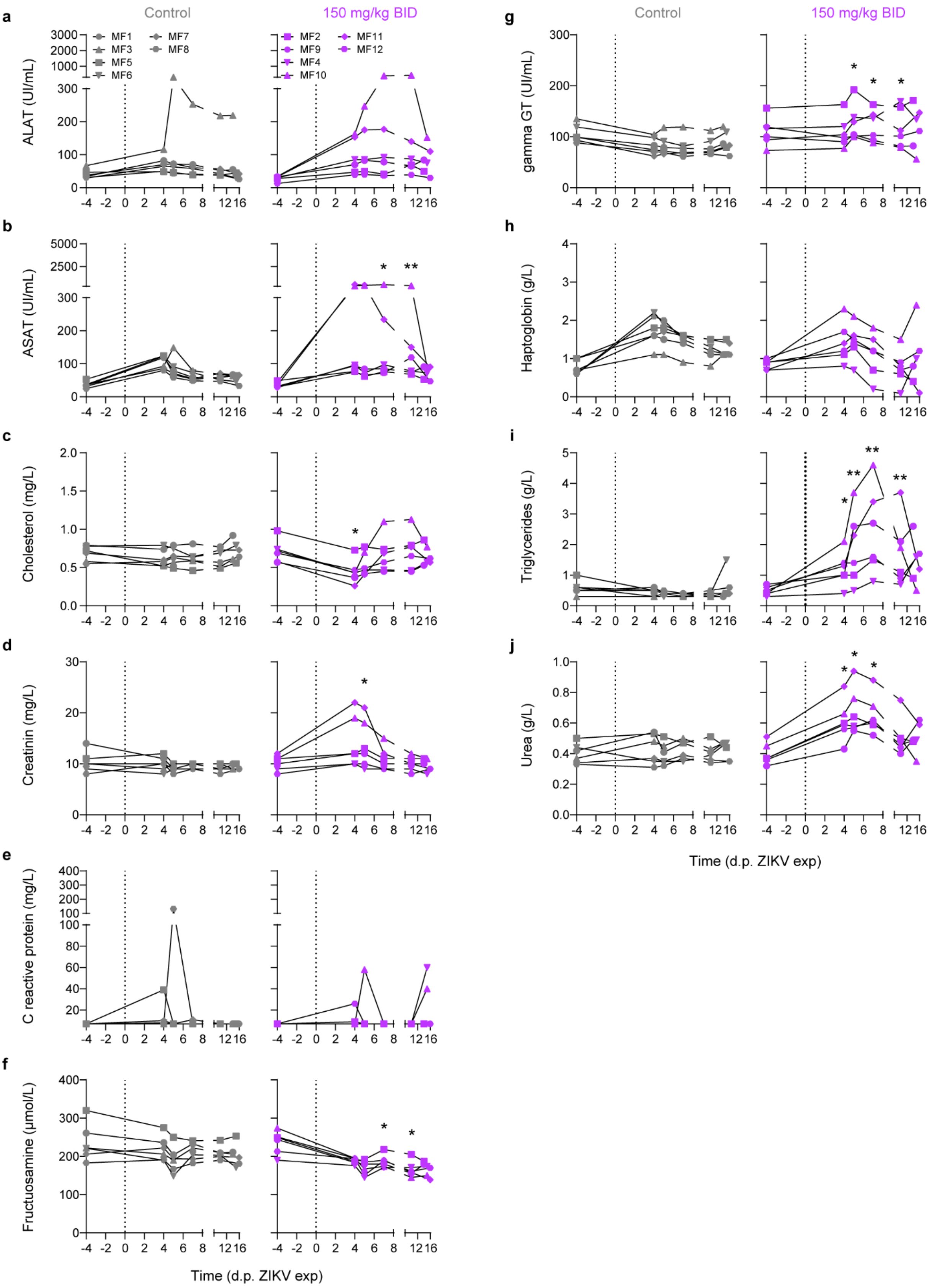

Figure S3. Biochemistry analysis of cynomolgus macaques after ZIKV infection.

a) ALAT, b) ASAT, c) cholesterol, d) creatinine, e) C reactive protein, f) fructosamine, g) gamma GT, h) haptoglobin, i) triglyceride and j) urea were analyzed in the plasma of all ZIKV infected animals. Grey: untreated; purple: 150 mg/kg BID. The asterisks indicate a significant difference between the control group and the FPV group. Parameters were compared between groups using the two-tailed non-parametric Mann-Whitney test. Source data are provided as a Source Data file.

Figure S4

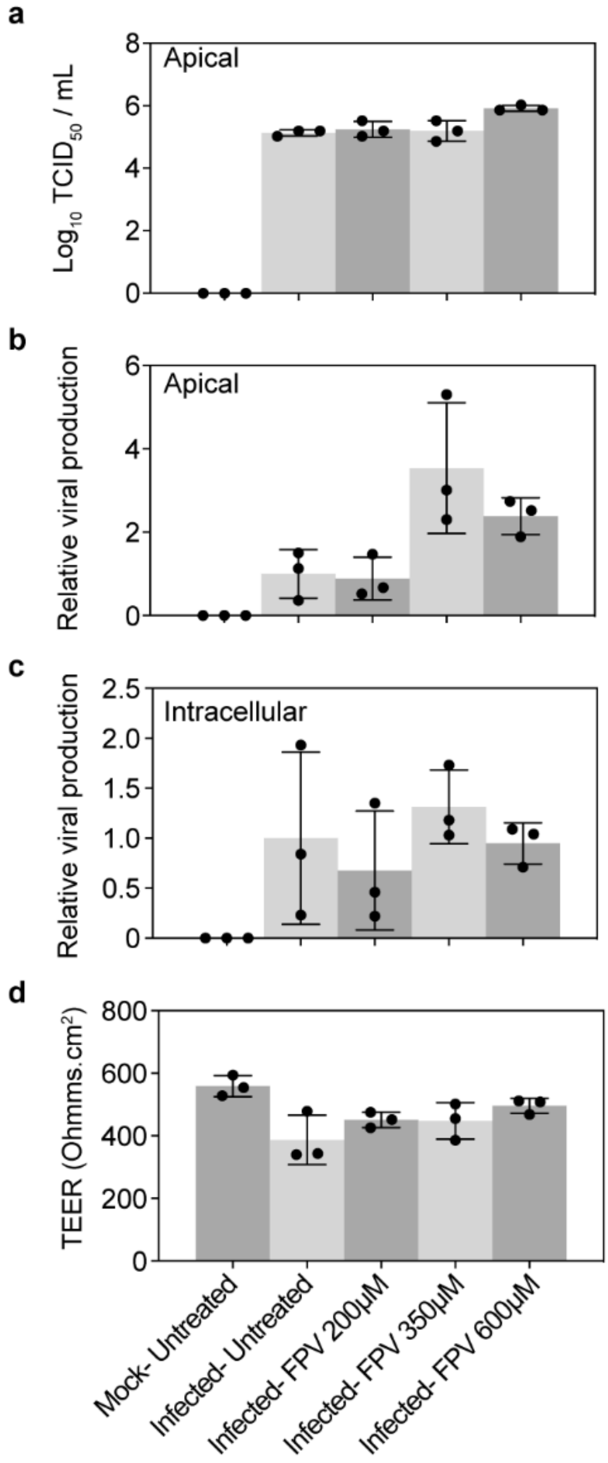

Figure S4. In vitro evaluation of the antiviral activity of PFV against SARS-CoV-2.

a) Infectious titers, b) apical and c) intracellular relative viral production and d) trans-epithelial resistance (TEER in Ohms.cm2) between the apical and basal poles in bronchial HAE at 48 h.p.i. MucilAir HAE reconstituted from human primary cells obtained from bronchial biopsies were provided

by Epithelix and maintained in air–liquid interphase. For infection experiments, apical poles were gently washed twice with warm OptiMEM medium and then infected directly with a 150-µl dilution of virus in OptiMEM medium, at a MOI of 0.1. For mock infection, the same procedure was performed using OptiMEM as inoculum. Results are expressed in relative viral production compared with the infected untreated control. Epithelial integrity was assessed by measure of variations in transepithelial electrical resistance (Δ TEER) and was expressed as Ohm.cm2. Data are presented as mean values +/- SD, n=3 biological replicates tested in duplicate.

Figure S5

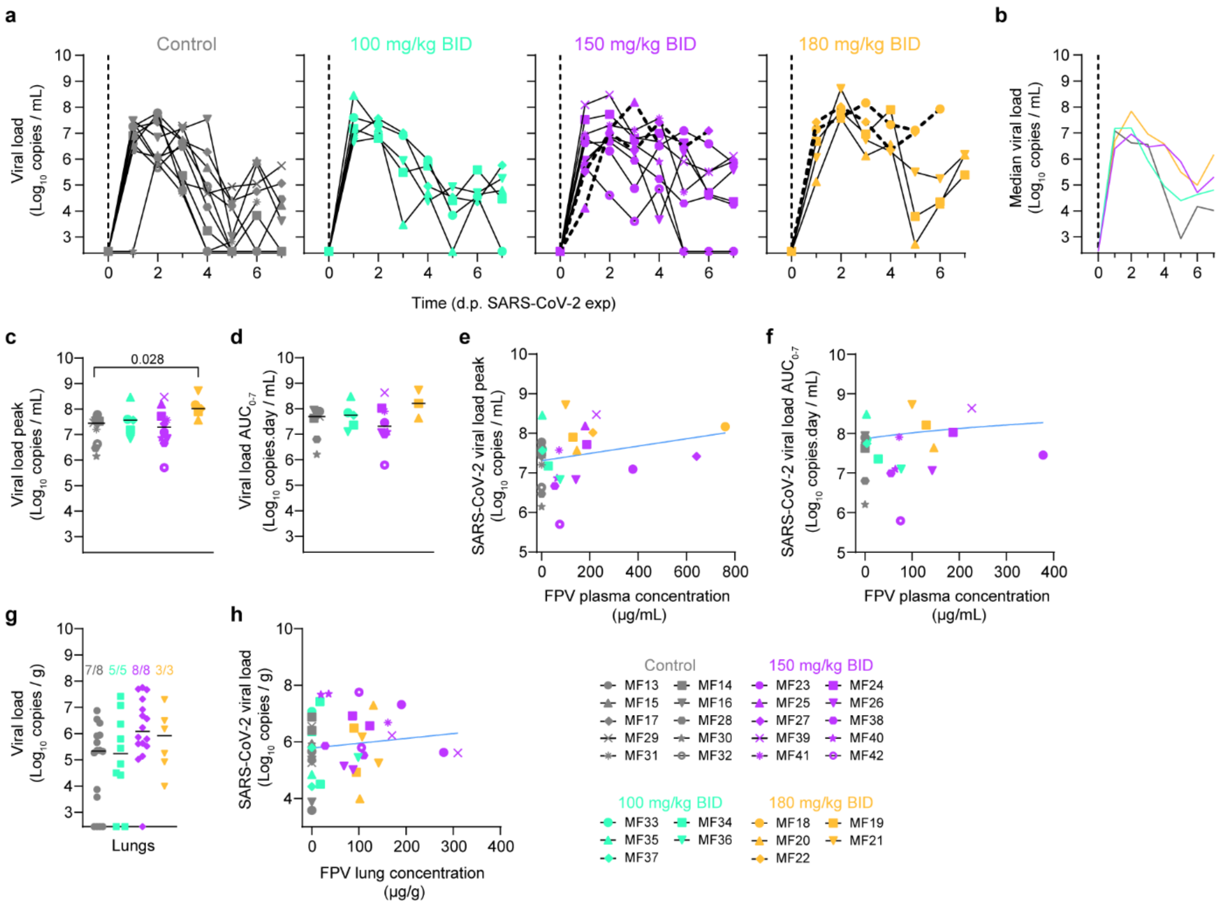

Figure S5. Viral loads in the tracheal fluids of SARS-CoV-2-infected cynomolgus macaques treated with FPV.

a) Individual tracheal viral loads determined by RT-PCR in all animals; b) Median tracheal viral load values observed in each treatment group; c and d) Viral kinetic parameters (peak and AUC viral load) during the first 7 days of infection. Median value is indicated by horizontal bar. Parameters were compared between groups using Kruskal-Wallis test following Dunn's multiple comparisons. e and f) Viral kinetic parameters (peak and AUC viral load between 0 and 7 dpi) according to geometric mean FPV plasma trough concentration. g) Viral load in lung biopsies sampled at euthanasia 7 d.p.e. The numbers indicated animals where viral load was detected in lung tissue. Median value is indicated by horizontal bar. h) Viral load in lung biopsies according to FPV lung concentration. A Spearman correlation test was performed to assess the association between drug concentration and viral kinetic parameters. Grey: untreated; cyan: 100 mg/kg BID; purple: 150 mg/kg BID; yellow: 180 mg/kg BID. Source data are provided as a Source Data file.

Figure S6

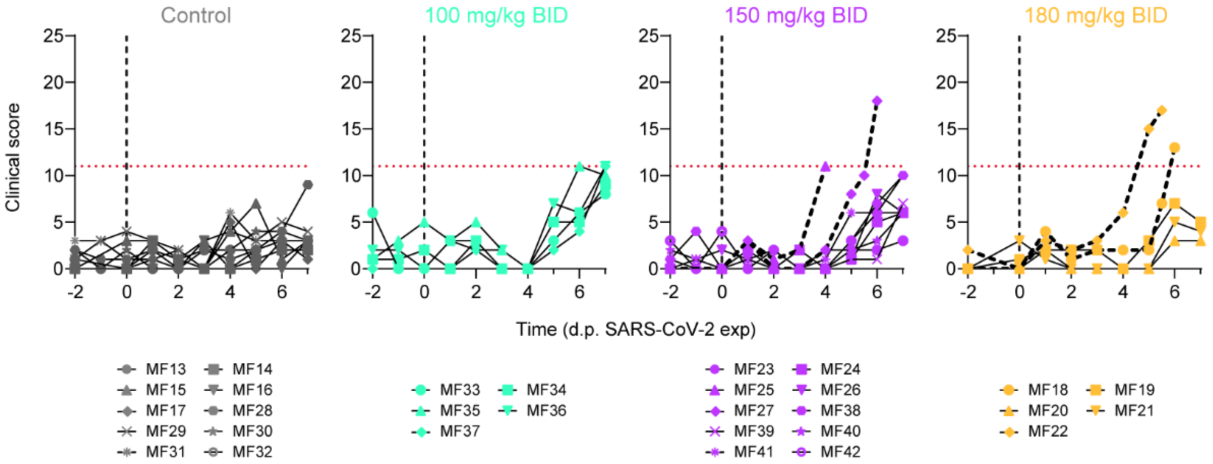

Figure S6. Clinical scoring of SARS-CoV-2-exposed cynomolgus macaques.

Clinical score was based on body weight evolution, rectal temperature, food/water consumption, activity, dehydration, respiration, heart rate and oxygen saturation. A score between 0 and 3 were allotted to each parameter. Sum of all recorded parameter were shown. Horizontal red dotted line indicated the human end point score. Source data are provided as a Source Data file.

Figure S7

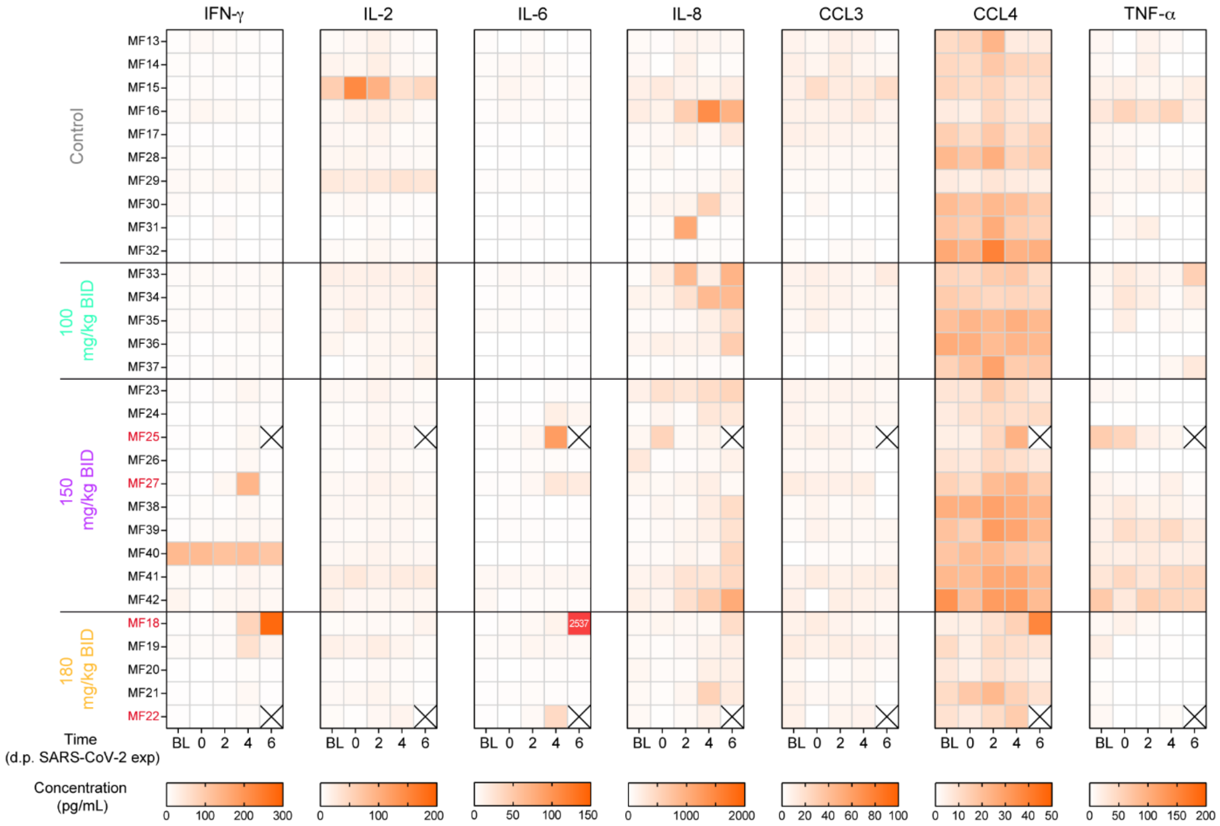

Figure S7. Cytokines and chemokines in the plasma of SARS-CoV-2-exposed cynomolgus macaques treated with FPV.

Heatmaps of the plasma concentrations of 7 cytokines and chemokines. Each column represents one cytokine or chemokine; the colour scale (in pg ml−1) is shown at the bottom. ‘X’ indicates that no measurement was feasible for this time point. Animals that were euthanized due to rapid deterioration of their clinical status were indicated in red. Source data are provided as a Source Data file.

Figure S8

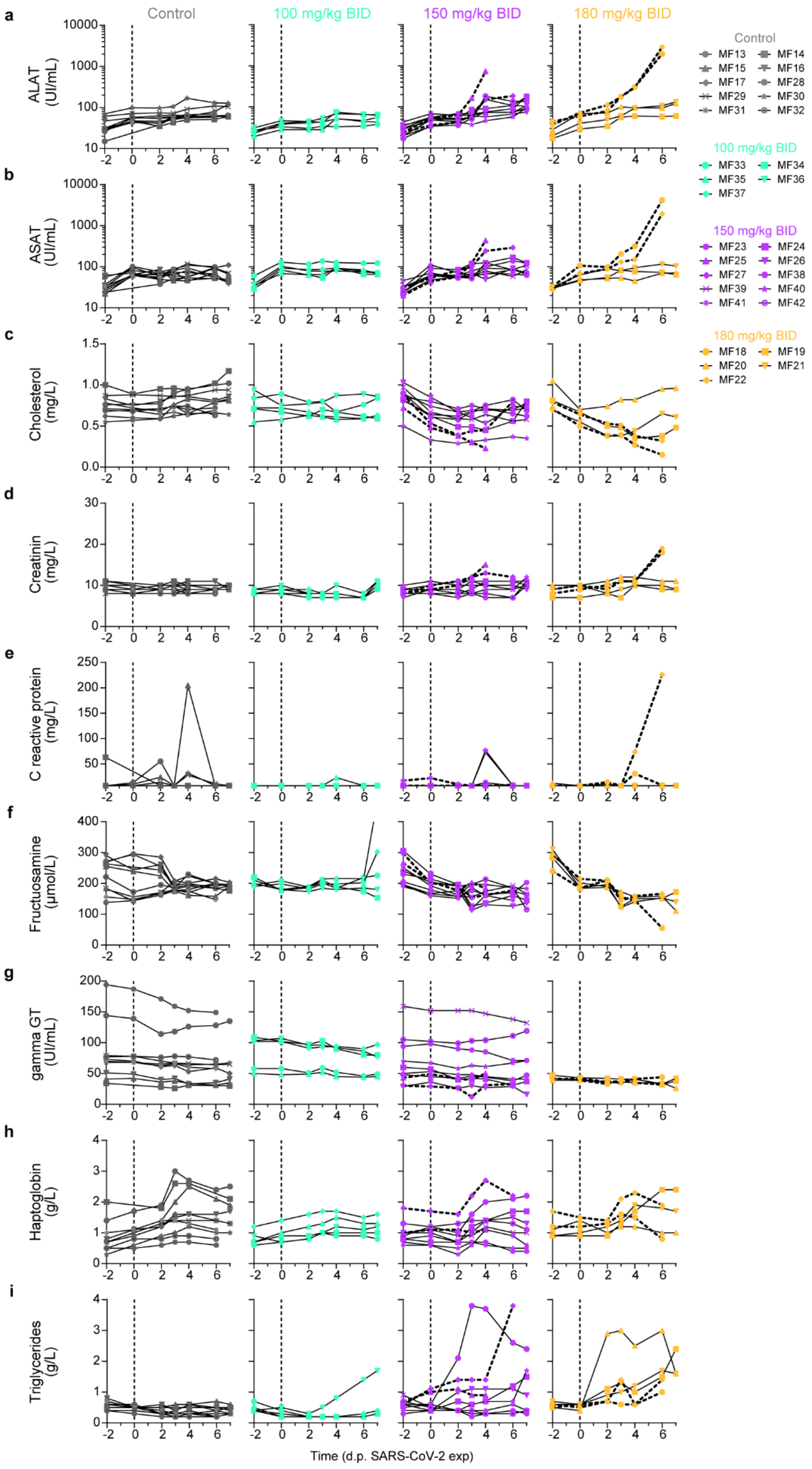
a
b
c
d
e
f
g
h
i
Control
100 mg/kg BID
150 mg/kg BID
180 mg/kg BID
ALAT (UI/mL)
ASAT (UI/mL)
Cholesterol (mg/L)
Creatinin (mg/L)
C reactive protein (mg/L)
Fructuosamine (µmol/L)
gamma GT (UI/mL)
Haptoglobin (g/L)
Triglycerides (g/L)
Time (d.p. SARS-CoV-2 exp)
Control
MF13 MF14
MF15 MF16
MF17 MF28
MF29 MF30
MF31 MF32
100 mg/kg BID
MF33 MF34
MF35 MF36
MF37
150 mg/kg BID
MF23 MF24
MF25 MF26
MF27 MF38
MF39 MF40
MF41 MF42
180 mg/kg BID
MF18 MF19
MF20 MF21
MF22

Figure S8. Biochemistry analysis of cynomolgus macaques after SARS-CoV-2 infection.

ALAT (a), ASAT (b), cholesterol (c), creatinine (d), C reactive protein (e), fructosamine (f), gamma GT (g), haptoglobin (h) and triglyceride (g) were analyzed in the plasma of all SARS-CoV-2 infected animals. Grey: untreated; cyan: 100 mg/kg BID; purple: 150 mg/kg BID; yellow: 180 mg/kg BID. Source data are provided as a Source Data file.

Figure S9

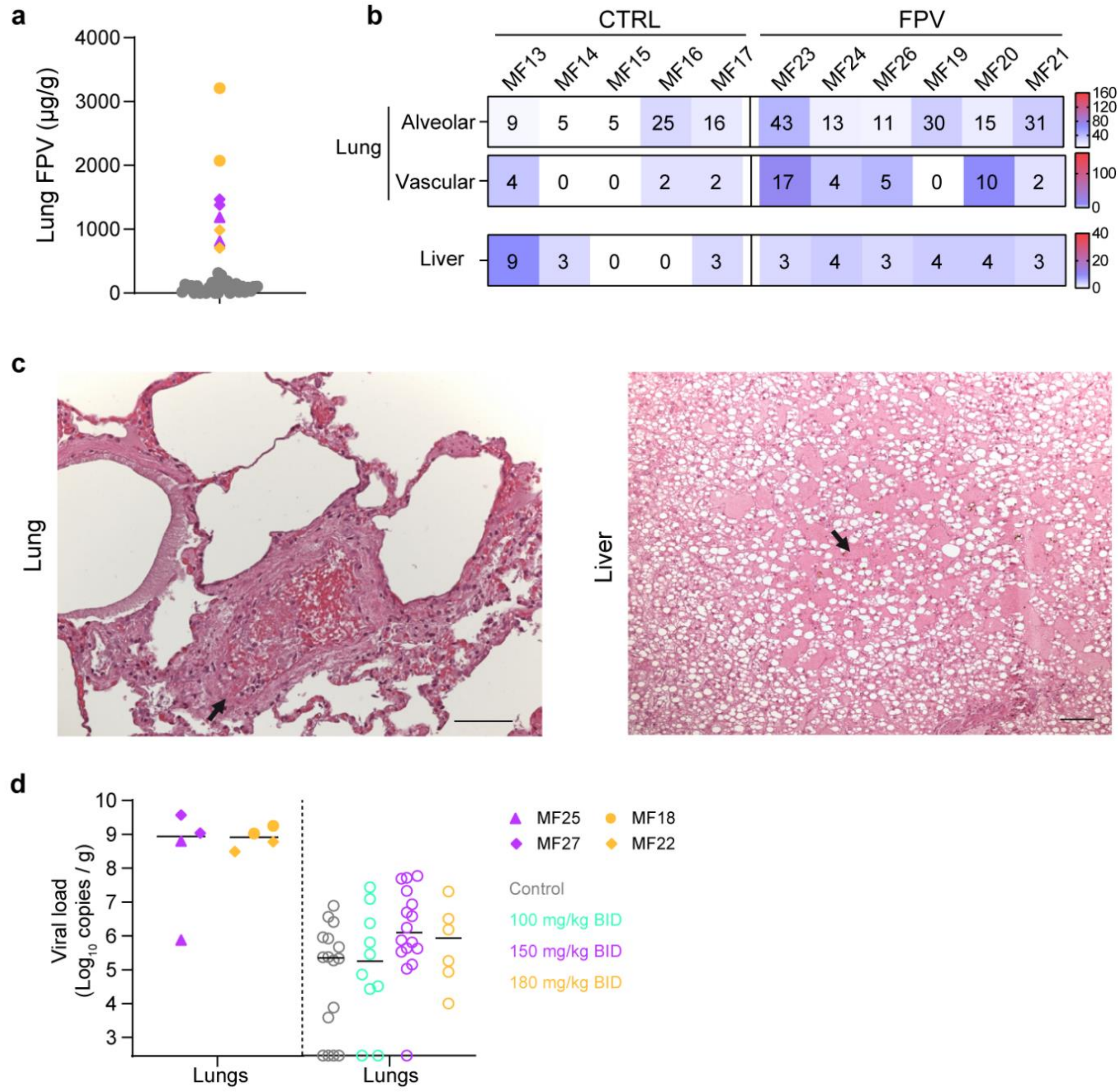

Figure S9. Histological analysis of tissues from SARS-CoV-2-exposed cynomolgus macaques.

a) FPV concentration in lung biopsies sampled at euthanasia. Two biopsies per animal were analyzed. Values for MF18, MF22, MF25 and MF27 are indicated in color according to the FPV dose (pink: 150 mg/kg BID and yellow: 180 mg/kg BID), and other FPV treated animals were indicated in grey. b) Tissue lesions and cell infiltrates were analyzed at necropsy at 7 dpe and histological score for lung (alveolar and vascular areas) and liver was showed in heatmap. c) Representative images of lung (left) and liver (right) of animals that were early euthanized. The black bars indicate 100µm. d) Viral load in lung biopsies sampled at euthanasia in MF18, MF22, MF25 and MF27 (left) and the rest of study's

NHPs (right). Two lung biopsies per animal were analyzed. Median value is indicated by horizontal bar.

Source data are provided as a Source Data file.

Figure S10

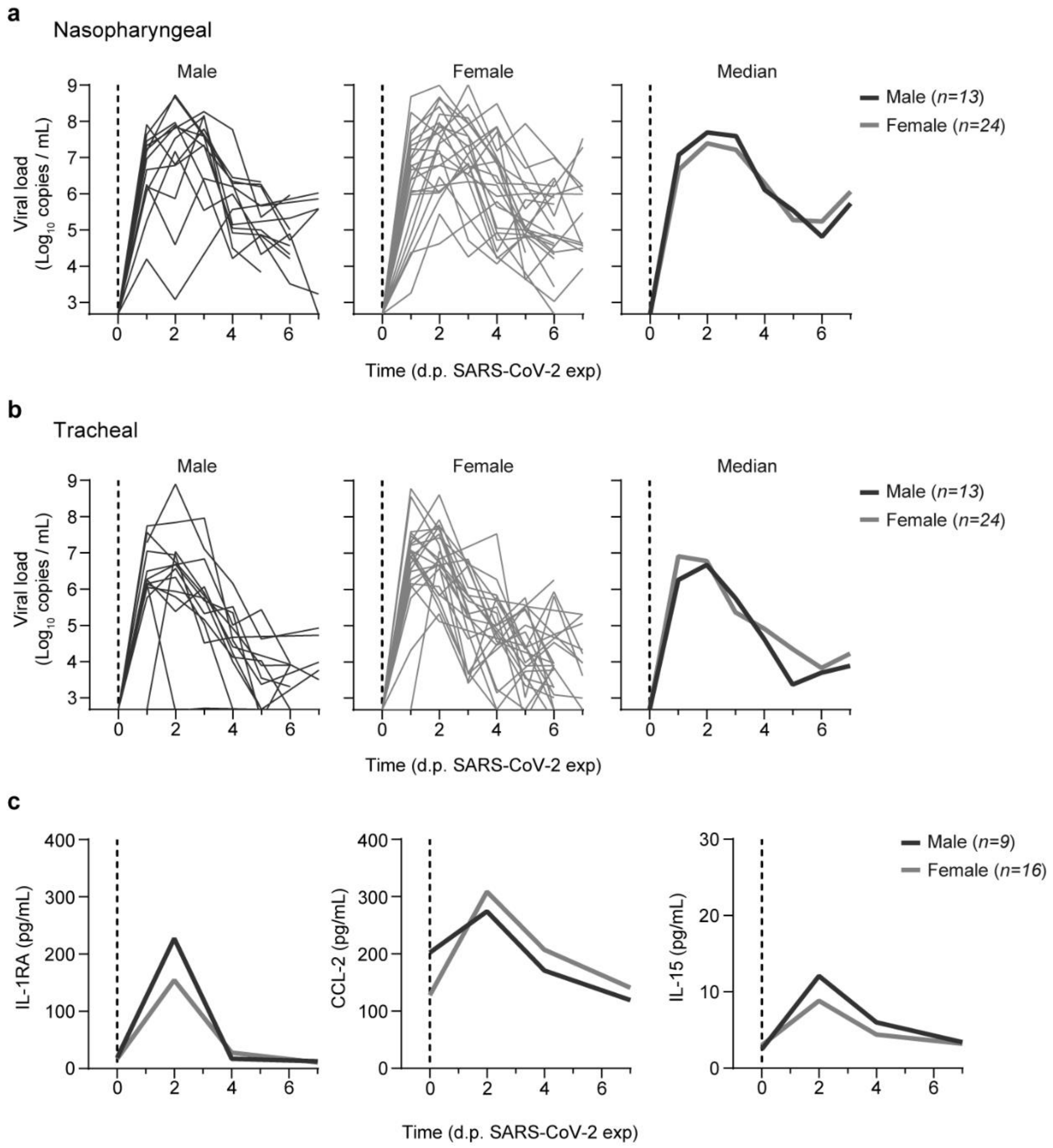

Figure S10. Comparison of SARS-CoV-2 viral loads between male and female cynomolgus macaques.

Individual nasopharyngeal (a) and tracheal (b) viral loads determined by RT-PCR in historical NHP exposed to 10^6 pfu of SARS-CoV-2 used as controls of therapeutic and vaccine trials (n=13 males and n=24 females). Median viral load values observed in male and female. Source data are provided as a Source Data file. c) Median plasma concentrations of IL1-RA, CCL-2 and IL-15 in historical male and female exposed to SARS-CoV-2.
