# Supplementary material for: Antiviral efficacy of favipiravir against Zika and SARS-CoV-2 viruses in non-human primates
Source: Nat Commun. 2022 Aug 30;13:5108. doi: 10.1038/s41467-022-32565-w (PMC9427089; doi:10.1038/s41467-022-32565-w)
Supplement: Supplementary file 3 — Reporting Summary [file 41467_2022_32565_MOESM3_ESM.pdf]

nature portfolio

Corresponding author(s): Roger Le Grand

Last updated by author(s): Jul 27, 2022

Reporting Summary

Nature Portfolio wishes to improve the reproducibility of the work that we publish. This form provides structure for consistency and transparency in reporting. For further information on Nature Portfolio policies, see our Editorial Policies and the Editorial Policy Checklist.

Statistics

For all statistical analyses, confirm that the following items are present in the figure legend, table legend, main text, or Methods section.

n/a	Confirmed	
☐	☒	The exact sample size (*n*) for each experimental group/condition, given as a discrete number and unit of measurement
☐	☒	A statement on whether measurements were taken from distinct samples or whether the same sample was measured repeatedly
☐	☒	The statistical test(s) used AND whether they are one- or two-sided<br>*Only common tests should be described solely by name; describe more complex techniques in the Methods section.*
☐	☒	A description of all covariates tested
☐	☒	A description of any assumptions or corrections, such as tests of normality and adjustment for multiple comparisons
☐	☒	A full description of the statistical parameters including central tendency (e.g. means) or other basic estimates (e.g. regression coefficient) AND variation (e.g. standard deviation) or associated estimates of uncertainty (e.g. confidence intervals)
☐	☒	For null hypothesis testing, the test statistic (e.g. *F*, *t*, *r*) with confidence intervals, effect sizes, degrees of freedom and *P* value noted<br>*Give P values as exact values whenever suitable.*
☒	☐	For Bayesian analysis, information on the choice of priors and Markov chain Monte Carlo settings
☒	☐	For hierarchical and complex designs, identification of the appropriate level for tests and full reporting of outcomes
☐	☒	Estimates of effect sizes (e.g. Cohen's *d*, Pearson's *r*), indicating how they were calculated

Our web collection on statistics for biologists contains articles on many of the points above.

Software and code

Policy information about availability of computer code

Data collection	Data was collected using classical Excel Files. Macaque data were stored in a Laboratory Information Management System called BatLab (V3.0).
Data analysis	Data was analyzed using GraphPad Prism v8, Microsoft Excel 2016 (16.0.5173.1000), 3DSlicer (Version 5), IntelliSpace Portal (V8), CFX Maestro (V2.2)

For manuscripts utilizing custom algorithms or software that are central to the research but not yet described in published literature, software must be made available to editors and reviewers. We strongly encourage code deposition in a community repository (e.g. GitHub). See the Nature Portfolio guidelines for submitting code & software for further information.

Data

Policy information about availability of data

All manuscripts must include a data availability statement. This statement should provide the following information, where applicable:

- Accession codes, unique identifiers, or web links for publicly available datasets
- A description of any restrictions on data availability
- For clinical datasets or third party data, please ensure that the statement adheres to our policy

Source data are provided with this paper. The data generated in this study are provided in the Source Data file.

Human research participants

Policy information about studies involving human research participants and Sex and Gender in Research.

Reporting on sex and gender	n/a
Population characteristics	n/a
Recruitment	n/a
Ethics oversight	n/a

Note that full information on the approval of the study protocol must also be provided in the manuscript.

Field-specific reporting

Please select the one below that is the best fit for your research. If you are not sure, read the appropriate sections before making your selection.

☒ Life sciences ☐ Behavioural & social sciences ☐ Ecological, evolutionary & environmental sciences

For a reference copy of the document with all sections, see nature.com/documents/nr-reporting-summary-flat.pdf

Life sciences study design

All studies must disclose on these points even when the disclosure is negative.

Sample size	Sample size was determined as the minimal number allowing non-parametric statistical analysis while complying with the 3Rs rule on reducing, replacing and refining the use of animals for scientific purpose.
Data exclusions	No data has been excluded from analysis.
Replication	Duplicates were performed for all measurments within each assay (RT-PCR, drug quantification, luminex and in vitro HAE system). All attemps were succefull. In vitro HAE system were performed in 3 biological replicates tested in duplicate.
Randomization	NHPs were randomly assigned in two experimental groups in ZIKV study, and in four experimental groups in SARS-CoV-2 study.<br>For experiments that do not include animals, this is not relevant as they only include animal samples that were all tested at the same time.
Blinding	For security reason, animal ID and experimental group are indicated on the housing cage, thus Animals care, clinical examination and sampling was not blinded because constrains associated to BSL3 containement.<br>Cynomolgus macaque viral loads, CT scoring, luminex, histological scoring, biochemistry analysis and drug quantification were determined blinded.

Reporting for specific materials, systems and methods

We require information from authors about some types of materials, experimental systems and methods used in many studies. Here, indicate whether each material, system or method listed is relevant to your study. If you are not sure if a list item applies to your research, read the appropriate section before selecting a response.

Materials & experimental systems

n/a	Involved in the study	
☐	☒	Antibodies
☐	☒	Eukaryotic cell lines
☒	☐	Palaeontology and archaeology
☐	☒	Animals and other organisms
☒	☐	Clinical data
☒	☐	Dual use research of concern

Methods

n/a	Involved in the study	
☒	☐	ChIP-seq
☒	☐	Flow cytometry
☒	☐	MRI-based neuroimaging

Antibodies

Antibodies used	Anti-SARS Nucleocapsid Protein; Novus Bio NB-56576; 2.5 µg/ml; Rabbit Polyclonal

Validation	Specificity of Anti-SARS Nucleocapsid Protein antibody was validated in house on negative (lung from uninfected Cynomolgus) and positive (lung from SARS-CoV-2 infected Cynomolgus macaque at 3 day post infection) slides. This antibody is also validated for immunostaining on manufacturer's webstite.

Eukaryotic cell lines

Policy information about cell lines and Sex and Gender in Research

Cell line source(s)	HAE : human primary cells obtained from bronchial biopsies; provided by Epithelix SARL (Ref. EP01MD, Geneva, Switzerland)
Authentication	Cells were not authenticated
Mycoplasma contamination	HAE are negative for mycoplasma contamination before shipment by provider.
Commonly misidentified lines (See ICLAC register)	HAE are primary cells and are not in ICLAC list.

Animals and other research organisms

Policy information about studies involving animals; ARRIVE guidelines recommended for reporting animal research, and Sex and Gender in Research

Laboratory animals	The study have included 42 cynomolgus macaques (Macaca fascicularis), aged 44-86 months (20 females and 22 males).
Wild animals	No wild animals were used in this study
Reporting on sex	Both males and females were included in these study. In our animal model, viral infection is similar in male and female cynomolgus macaques. We are also providing viral kinetics and cytokine profile in our historical NHP infected with SARS-CoV-2 used as controls of therapeutic and vaccine trials.
Field-collected samples	No field-collected samples were used in this study.
Ethics oversight	Cynomolgus macaques (Macaca fascicularis), aged 44-86 months (20 females and 22 males) and originating from Mauritian AAALAC certified breeding centers were used in this study. All animals were housed in IDMIT infrastructure facilities (CEA, Fontenay-aux-roses), under BSL-2 and BSL-3 containment when necessary (Animal facility authorization #D92-032-02, Prefecture des Hauts de Seine, France) and in compliance with European Directive 2010/63/EU, the French regulations and the Standards for Human Care and Use of Laboratory Animals, of the Office for Laboratory Animal Welfare (OLAW, assurance number #A5826-01, US). The protocols were approved by the institutional ethical committee "Comité d'Ethique en Expérimentation Animale du Commissariat à l'Energie Atomique et aux Energies Alternatives" (CEtEA #44) under statement numbers A16-013 and A20-011. These studies were authorized by the "Research, Innovation and Education Ministry" under registration numbers APAFIS#4079-2016021212132792v3 and APAFIS#24434-2020030216532863v1.

Note that full information on the approval of the study protocol must also be provided in the manuscript.
